# Testing for Partial RhD with a D-Screen Diagast Kit in Moroccan Blood Donors with Weak D Expression

**DOI:** 10.1155/2014/204301

**Published:** 2014-10-28

**Authors:** Z. Kabiri, M. Benajiba, K. Hajjout, N. Dakka, H. Bellaoui

**Affiliations:** ^1^Centre National de Transfusion Sanguine, rue Lamfadel Cherkaoui, Madinat Al Irfane, P.O. Box 180, 10000 Rabat, Morocco; ^2^Departement de Biologie, Faculté des Sciences, University MDV Rabat Agdal, 1014 Rabat, Morocco; ^3^Centre Regional de Transfusion Sanguine, rue Lamfadel Cherkaoui, Madinat Al Irfane, P.O. Box 180, 10000 Rabat, Morocco

## Abstract

The aim of this study was to search for the partial D phenotype in Moroccan blood donors with weak D expression. The study included 32 samples with weak D phenotype, and partial D category red blood cells were detected with the D-Screen Diagast kit, which consists in 9 monoclonal anti-D antibodies specific for the most common categories of partial D. Among the 32 samples studied, we identified 13 specific reactions to a partial D antigen (3 DVI, 2 DVa, 2 DIII^(a,b,c)^, and 6 DVII), with 8 reactions suggesting a weak D and 11 reactions providing no formal argument in favor of a partial D antigen. This work can be used to validate the performance of the anti-D reagent and to improve the safety of transfusion of red blood cells from donors expressing the partial D antigen by integrating the finding into the recipient file with a recommendation concerning the appropriate care.

## 1. Introduction

Rhesus is one of the most important and clinically significant blood group systems. D antigen (ISBT 004.001; RH1) is the most immunogenic and clinically important of this system because of the ability of anti-D to cause transfusion reactions and hemolytic disease of the foetus and newborn. The Rh system was described for the first time in 1939 and is now considered to be a mosaic of epitopes (antigenic determinants). Partial D variants lack one or more epitopes of D antigen while weak D variants have all epitopes present but express a significantly reduced amount of D antigen per red blood cell and are usually identified by the indirect antiglobulin test (IAT). Partial D and weak D are the most commonly found D variants. Individuals whose red blood cells do not carry all the parts of the D mosaic can, when exposed to the full D antigen, produce anti-D alloantibodies directed against one or more of the missing epitopes, thus defining the phenotype “partial D.” Loss of D epitopes is associated with either gene rearrangements or point mutations affecting extracellular portions of the RhD protein [1, 2]. The great diversity of D variants (weak D and partial D) explains the discrepancies noted between two serological determinations and the lack of reactivity of certain variants by serology [3]. It is very important to identify a donor having a D variant (weak D or D partial D) since in some instances these red blood cells can trigger an immune response if transfused to a recipient who is D negative.

The study of D variants in blood donors for immunohematological qualification was little studied in Morocco and thus in order to provide elements of information on the prevalence of weak D and the identification and the frequency of certain D variants, our earlier study [4], conducted among Moroccan blood donors in which we tested 23098 samples of Moroccan blood donors to determine the incidence of weak D phenotype, showed that 9.5% (2204) of the donors were RhD negative and the weak D phenotype was detected in about 0.4%. Therefore, the purpose of this present study was to identify the partial D phenotype among 32 Moroccan blood donors with weak D expression collected in total of 59693 samples using the D-Screen Diagast kit of nine anti-D monoclonal antibodies and to guide immunohematologist in the resolution of serological difficulties (discrepancies) in order that the correct D antigen status can be assigned and in choosing the right strategy adapted to the Moroccan population in terms of prevention of alloimmunization and fetal-maternal transfusion.

## 2. Subjects and Methods

### 2.1. Subjects

This study is concerned with 32 samples of Moroccan blood donors with D variant phenotype, collected in total of 59693 blood donors at the National Blood Transfusion Centre of Rabat, for over one year.

### 2.2. Methods

#### 2.2.1. Determination of the RhD Phenotype and Weak D Phenotype

The RhD phenotype was performed with an Olympus pk7300 analyzer. We used the anti-D IgM Diagast (clone P3 × 61) for the first determination and the anti-D IgG Diagast TOTEME (clones P3 × P3 × 61 + 21223B10 + P3 + P3 × 290 × 35) for the second determination. Weak D expression of the D antigen was performed systematically for all RhD negative blood donors using the indirect Coombs test. Red blood cells of blood donors with the weak D phenotype were subsequently studied, using the hemagglutinin tube technique, with a panel of nine monoclonal antibodies anti-D IgM and IgG of the D-Screen Diagast kit. These reagents were selected for their ability to define specific reaction profiles of the most frequent partial D following the classifications DII, DIIIa, DIIIb, DIIIc, DIVa, DIVb, DVa, DVI, DVII, DFR, DBT, DHAR, and DHMi.

- For the anti-D antibody of IgM type, the reaction used a direct hemagglutinin test tube, while for the D-type anti IgG antibody, the reaction consisted in the indirect Coombs test tube of antiglobulin human (AHG) Mestria IGG or IGG + C3D.

## 3. Results

- In total of 59693 samples analyzed by Olympus pk7300, 6612 of the donors were RhD negative and the weak D phenotype was detected in about 0.5% (*n* = 32).
- Screening for Rh partial D with the D-Screen Diagast kit of nine anti-D monoclonal antibodies allowed us to identify in the 32 samples the following phenotypes: (Table 1)
  - 13 (40.7%) specific reactions to the partial D antigen defined as follows: 3 (9.37%) DVI, 2 (6.25%) DV^a^, 2 (6.25%) DIII^(a,b,c)^, and 6 (18.75%) DVII.
  - 8 (25%) reactions positive only with IgM anti-D and with most performed IgG anti-D, suggesting a weak D antigen.
  - 11 (34.37%) positive reactions with all the kit reagents, which does not provide formal arguments in favor of a partial D antigen.

## 4. Discussion

The distinction between D positive and D negative red blood cells is not always obvious in the case of D variants [5]. The partial D and weak D phenotypes give discrepant results when using different marketed monoclonal anti-RhD reagents [6, 7]. Depending on the presence or absence of D epitopes on red blood cells, those with partial D may be typed as D positive or negative with commercial anti-D reagents [8].

In our study, which consisted of a population of 32 weak D phenotype donors, we identify, 13 samples with a partial D phenotype (3 DVI, 2 DVa, 2 (DIIIa, DIIIb, DIIIc), and 6 DVII), identified with the D-Screen Diagas kit. However, we cannot provide any conclusions regarding the frequency of these variants in the Moroccan population due to the limited number of samples obtained in our study (32 weak D), because the weak D phenotype is infrequent in Morocco (0.4%) [4].

The frequency of partial D varies from one ethnic group to another, and in the Caucasian population the frequency of variants DVI is 1 : 6.200 [9] and among black individuals the frequency of certain partial D variants (DIIIa, DIIIb, and DIVa) is relatively high [10, 11]. In addition, it is very important to identify and assess the frequency of partial D variants, in particular the partial DVI variant, because this category is characterized by a reduced number of antigenic sites per cell. These variants can be typed by serology as false negatives (DVI type I: 300 antigens/cell) [12, 13]. On other hand the absence of detection of certain variants by serological techniques has been reported by several authors. Engelfriet et al. [14] calculated that in Southern California alone each year the red cells from at least 120 weak D or DEL donors, typed D negative serologically, are transfused to D negative recipients. Yet, no cases of unexpected anti-D immunization have been recorded, because very weak D or Del cells, with a very small number of D sites, only very rarely induce a primary anti-D immune response in D negative recipients and are only found to be D positive by RHD genotyping.

In our study using D-Screen Diagast kit containing a panel of nine monoclonal anti-D, we identified 13 (40.7%) reactions of partial D phenotype, 8 (25%) reactions of weak D phenotype, and 11 (34.37%) indeterminate reactions. However, based on our findings in this study, this kit was not very useful for the identification of most D variants. Therefore it is very important to identify these samples of weak D and the 11 indeterminate phenotypes in the further work, by using another kit of D partial or by analyzing the samples with PCR and sequencing, because they could not exclude the possibility for partial D.

Although it is difficult in blood banks to differentiate between partial D and weak D, it is important to identify a donor as having a D variant as the red cells of such a donor could elicit an immune response if transfused to a D negative recipient.

In Europe, DVI is most frequently associated with alloimmunization. Therefore, an unexpected feature of DVI type III was its almost normal number of RhD proteins per cell [15]. Furthermore to limit anti-D immunization for DVI recipient, the strategy of screening was essentially RhD antigen density-based transfusion strategy that is today considered wasteful, as it became apparent that most weak D (types 1 and 2) may be safely transfused RhD positive such that the goal of Wanger et al. [16]. The wastage might be reduced by lowering of the weak D threshold for RhD negative transfusion. However, this measure would trigger RhD positive transfusion in partial D like DVI type III, while still many RhD negative units would be transfused to weak D patients not requiring RhD negative transfusion. In this context, a strategy based on two monoclonal anti-D that do not react with DVI is advantageous [9]. This RhD epitope-based transfusion strategy abandons RhD antigen density as the trigger for RhD negative transfusions and became mandatory in Germany in 1996 [17]. For donor typing, weak D is considered Rhesus positive [18]. DVI type III proved that DVI erythrocytes may carry rather high RhD antigen densities. The threshold of RhD antigen density and RhD epitopes that most likely cause anti-D immunization is not fully established [15].

Based on our findings in this study, it seems appropriate to use molecular test in the next study which will identify the majority of D variants in our population and also resolve serological discrepancies. On the other hand, it is important to follow a strategy that consists firstly in selecting a monoclonal anti-D antibody that does not recognize DVI category for potential recipients of blood products. This should lead to better prevention of alloimmunization and to optimization of fetal-maternal transfusion [1]. Secondly, we need to increase the number of samples in order to better study the frequency of the most frequent partial D phenotypes in our population to guide immunohematologist in resolving serological discrepancies and in choosing the right strategy adapted to our population in terms of prevention of anti-D alloimmunization.

## 5. Conclusion

In conclusion, our study identified D variants (weak D and partial D categories) of the antigen D. This work can be used to validate the performance of the anti-D reagent and to improve the safety of transfusion of red blood cells from donors expressing the partial D antigen by integrating the finding into the recipient file with a recommendation concerning the appropriate care.

## Figures and Tables

**(a):** 

Number	Clone	Type	Two reactions DIIIa DIIIb DIIIc	Two reactions (Dva)	Three reactions (DVI)	Six reactions (DVII)	Eight reactions (weak D)							
1	HM10	IgM	+	+	−	+	+	+	+	+	+	+	+	+
2	HM16	IgG	+	+	−	+	−	−	+	−	+	+	−	−
3	P3 × 61	IgM	+	+	−	+	+	+	+	+	+	+	+	+
4	P3 × 35	IgG	+	−	−	+	+	+	+	−	−	+	−	+
5	P3 × 21211F1	IgM	+	+	−	−	+	+	+	+	+	+	+	+
6	P3 × 21223B10	IgM	+	+	+	+	+	+	+	+	+	+	+	+
7	P3 × 241	IgG	+	−	−	+	−	−	−	+	+	−	+	+
8	P3 × 249	IgG	+	+	−	+	−	+	+	−	−	−	−	−
9	P3 × 290	IgG	+	+	+−	+	+	−	+	−	+	−	−	+

Frequency			6.25%	6.25%	9.37%	18.75%	25%							

**(b):** 

Number	Clone	Type	11 indeterminate reactions (34.37%)										
1	HM10	IgM	+	+	+	+	+	+	+	+	+	+	+
2	HM16	IgG	+	++	+	+−	++	++	++	++	++	+	++
3	P3 × 61	IgM	−	+	+	+	+	+	+	+	+−	+	+
4	P3 × 35	IgG	+	+	+	+	+−	+	+−	+	+	+	+
5	P3 × 21211F1	IgM	+	+	++	+	+	+	+	+	+	+	+
6	P3 × 21223B10	IgM	++	+	+	+	+	+	+	+	+	+	+
7	P3 × 241	IgG	+	+	+	+	+	+	++	+	+	+	−
8	P3 × 249	IgG	+	+−	+	+	+	+−	+	−	+	+−	+
9	P3 × 290	IgG	+	+	−	+	+	+	+	+	+	+	+

+: positive reaction.

−: negative reaction.

+−: positive or negative reaction.
